# Recent Advances in Cell Penetrating Peptide-Based Anticancer Therapies

**DOI:** 10.3390/molecules24050927

**Published:** 2019-03-07

**Authors:** Justine Habault, Jean-Luc Poyet

**Affiliations:** 1 INSERM U976, Institut de Recherche St Louis, 1 avenue Claude Vellefaux, 75010 Paris, France; justine.habault@inserm.fr; 2 Université Paris Diderot, Sorbonne Paris Cité, 75013 Paris, France; 3 c-Dithem, Inserm Consortium for Discovery and Innovation in Therapy and Medicine, 75013 Paris, France

**Keywords:** cell-penetrating-peptides, protein transduction domains, cancer

## Abstract

Cell-penetrating-peptides (CPPs) are small amino-acid sequences characterized by their ability to cross cellular membranes. They can transport various bioactive cargos inside cells including nucleic acids, large proteins, and other chemical compounds. Since 1988, natural and synthetic CPPs have been developed for applications ranging from fundamental to applied biology (cell imaging, gene editing, therapeutics delivery). In recent years, a great number of studies reported the potential of CPPs as carriers for the treatment of various diseases. Apart from a good efficacy due to a rapid and potent delivery, a crucial advantage of CPP-based therapies is the peptides low toxicity compared to most drug carriers. On the other hand, they are quite unstable and lack specificity. Higher specificity can be obtained using a cell-specific CPP to transport the therapeutic agent or using a non-specific CPP to transport a cargo with a targeted activity. CPP-cargo complexes can also be conjugated to another moiety that brings cell- or tissue-specificity. Studies based on all these approaches are showing promising results. Here, we focus on recent advances in the potential usage of CPPs in the context of cancer therapy, with a particular interest in CPP-mediated delivery of anti-tumoral proteins.

## 1. Introduction

According to the World Health Organization, cancer (or malignant neoplasm) is the second leading cause of death worldwide (about 1 death in 6). This term regroups a large number of diseases all characterized by an abnormal division of cells that can invade nearby tissues and other parts of the body through the blood and lymph system (source: National Institutes of Health (NIH)—National Cancer Institute). Much effort has been dedicated to finding novel therapies against cancer in the past decades, but many obstacles must be overcome, such as drug-resistance, toxicity towards non-malignant cells and side effects, and inefficiency of drug delivery [1]. For the latter, one cause can be the inaptitude of pharmaceutical compounds to cross the plasma membrane, a semi-permeable hydrophobic barrier that insures the integrity of cells [2]. Hence, several recent studies focus on the development of alternative drug delivery systems, such as viral based-vectors, nanoparticles, or cell-penetrating peptides (CPPs) that enhance cell internalization [3,4,5]. CPPs, also known as protein transduction domains (PTDs), are defined as short peptides (less than 30 residues) with the ability to cross biological membranes in an energy-dependent or -independent manner [5]. In 1988, Joliot and his team discovered the Antennapedia homeodomain protein, a drosophila transcription factor able to enter nerve cells and control neural morphogenesis genes [6]. Shortly after, Derossi and colleagues identified the first CPP by demonstrating that the third helix of the Antennapedia homeodomain protein, named Penetratin, was the minimal sequence necessary for cell entrance [7]. Since then, more than 1700 CPPs have been characterized and listed in the CPPsite 2.0 database [8]. They have been experimentally validated for in vitro and in vivo delivery of small or large (up to 120 kDA) bioactive cargo inside cells [7,9,10,11,12,13,14,15,16,17,18,19,20,21,22]. Several complete reviews describe different ways to classify CPPs, for example, depending on their origin (protein-derived, synthetic, or chimeric), their physicochemical properties (cationic, amphipatic, or hydrophobic), or their uptake mechanism [5,16,23,24,25,26,27,28,29,30,31,32,33,34]. A non-exhaustive list of well-known CPPs is shown in Table 1.

It is also possible to sort CPPs depending on their range of applications. Indeed, thanks to their unique ability to transport various cargos inside cells with limited toxicity [28], CPPs are now considered as a powerful tool for both fundamental biology and medical applications. For instance, they can deliver contrast agents, such as Quantum dots [35] or metal chelates [36], for cell imaging purposes. Moreover, they can transport nucleic acids (siRNA, antisense oligomers, plasmids, decoy DNA), for which intracellular delivery is often limited by high molecular weight and negative charges, making the regulation of gene expression easier [29]. Finally, they can mediate drug delivery, ranging from nanoparticles to therapeutic proteins, and have been successfully used in a number of in vitro and in vivo studies. Importantly, while CPPs are able to cross cellular membranes, several studies demonstrated that most of them cannot cross the blood-brain barrier (BBB), which protects the central nervous system from toxicity. More than 25 CPP-conjugated drugs are under clinical development in applications as diverse as inflammation [37,38], pain [39,40,41], cancer [42], heart diseases [43,44,45], and aging [46]. An up-to-date version of completed phase I to III clinical trials is shown in Table 2.

The diversity of pathways and cell types targeted demonstrates the boundless potential of CPP-based therapies. Their success comes not only from their great intracellular delivery performance but also from their versatility; they are simple to synthesize, to modify, and to improve. However, to date, there are still no FDA approved CPP-conjugated drugs and several clinical trials have been discontinued. Among the reasons we can quote are: (1) in vivo stability issues, due to frequent susceptibility to proteolytic degradation [47]; (2) immunogenicity issues; (3) poor efficiency due to the drug’s failure to escape from endosomes after being internalized by cells; (4) toxicity due to the degradation of excipients; and (5) toxicity or poor efficiency due to the CPP’s lack of specificity. Indeed, while it is known that cationic CPPs interact with glycosaminoglycans [48], it is not yet established whether they can bind to specific membrane receptors. A wide biodistribution of CPP-conjugated drugs can lead to a reduction of drug efficiency due to a lower local concentration. Cost must also be considered, as well as risks of off-target effects. Hence, maximizing tumoral cell targeting while sparing normal cells is crucial.

In view of these challenges, we here present a review of the methods for CPP optimization and the latest promising CPP-based anticancer therapeutic strategies, especially CPP-protein complexes.

## 2. CPPs as Molecular Carriers in Cancer

### 2.1. CPP-Cargo Complexes Internalization

CPPs and their molecular cargoes can be bound using two different approaches. One way is to link CPPs and cargoes non-covalently using electrostatic interactions. For example, amphipathic peptide carriers, such as MPG and Pep-1, can form complexes with cargoes without the need for any crosslinking or chemical alterations [30,31,49,50]. The other way, which is more frequent, is to make a covalent connection between the two molecules. This method has been extensively used and has shown good results with carriers as varied as Penetratin, TAT, or Polyarginines [31,51,52].

**Figure 1:**
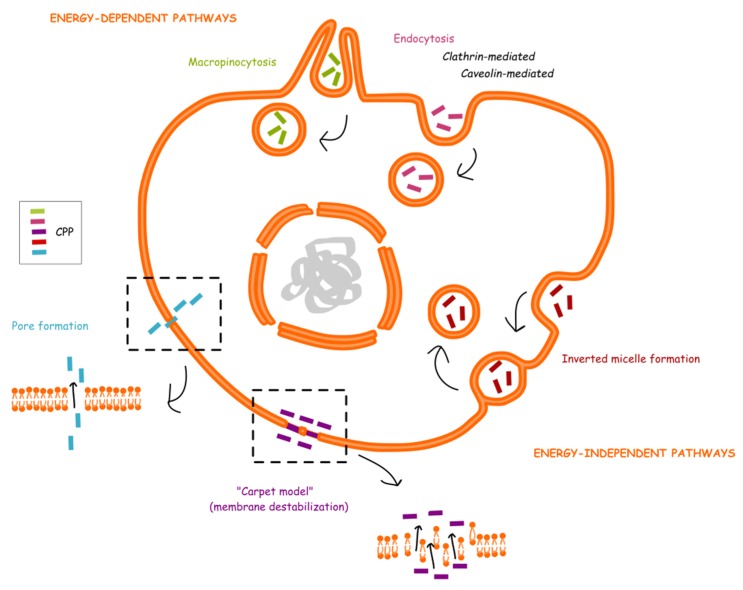
CPP translocation mechanisms.

Several models have been proposed for CPP internalization, but the exact mechanism remains poorly understood. However, evidence supports that both direct translocation (energy-independent mechanism) and endocytosis (energy-dependent) are involved in the cellular uptake process [27,32,53]. For direct translocation, at least three models have been described: the inverted micelle model due to the lipid bilayer interaction with the peptide [54], the pore formation [55], and the “carpet” model (membrane destabilization) [56]. For endocytosis, caveolae, and clathrin independent or dependent pathways have been reported [57,58,59], as well as macropinocytosis [60,61,62]. A summary of CPP internalization models is shown in Figure 1. It also seems that many factors, such as experimental conditions (temperature and pH), cell type, nature of CPP, nature of cargo, amount of CPP, and cargo are influencing the translocation process, as well as endosomal escape efficiency [27,32,53].

### 2.2. Delivery of Chemotherapeutic Agents

In the past few years, a handful of studies suggested that linking chemotherapeutic agents to CPPs could increase their efficiency by promoting intracellular delivery. For instance, Szabo et al. showed that methotrexate (MTX) covalently attached to the N-terminal of CPPs (Penetratin or R8) via peptide bons more-efficiently enter breast cancer cells, especially MTX-resistant cells [63]. More recently, Movafegh and co-workers demonstrated that poly-L-arginine could also increase the cellular uptake of doxorubicin (DOX), as well as its cytotoxicity towards human prostate cancer cells [64]. Likewise, doxorubicin’s stable conjugation via covalent bond with other CPPs, such as TAT (transactivator of transcription) and Penetratin, as well as the novel KRP, enhances its therapeutic effect [65,66,67]. In particular, KRP-DOX accumulates in the tumors by the enhanced permeability and retention (EPR) effect and KRP, thanks to its two nuclear localization sequences, leads the drug to the nucleus. Another example is the combination of Paclitaxel (PTX) with TAT—the covalent drug-peptide complexes display a greater anti-tumoral activity against human lung and breast cancer cell lines. Based on their uptake experiment results, the authors speculate that PTX-TAT may enter the tumor cells via an energy-independent, direct translocation pathway [68]. Finally, the experiment by Izabela et al. indicated that Transportan 10 (TP10) significantly improves the anti-cancer effect of cisplatin in human cervical tumoral and osteosarcoma cells compared to the drug alone. In this setting, non-covalent carrier-cargo complexes are formed with a metal-affinity-based linkage, and TP10-cisplatin seems to enter tumor cells via non-endocytic concentration-dependent pathways [69]. All these reports and several others provide evidence that the use of CPP for the delivery of chemotherapy may be therapeutically valuable thanks to enhanced pharmaceutical features.

### 2.3. Delivery of Nucleic Acids

Another field of medicine that could benefit from the use of CPP is gene therapy. Gene therapy represents a real hope for the prevention and treatment of many diseases, including cancer. However, nucleic acids are large and hydrophilic, meaning they are not able to cross cell membranes [29]. To overcome this problem, different vectors have been used but most of them, especially viral particles, display a high toxicity [70]. It is now known that cationic CPPs, such as TAT [61] or hPP10 [70], can be bound in a covalent or non-covalent fashion to unprotected nucleic acids (plasmid DNA and small interfering RNA). In the context of cancer, Lee and colleagues recently investigated the ability of a new CPP, BR2, to bind to VEGF siRNA and inhibit the expression of target genes [71]. VEGF is a key mediator of angiogenesis, a hallmark of cancer [72]. Their experiments suggest that cationic BR2 can form electrostatic interactions with anionic VEGF-siRNA thanks to the addition of nitrogen/phosphate (ratios above 4) and that the positively charged complexes of BR2-siRNA can enter the cytoplasm of cells to promote knockdown of target genes. Another interesting work has been performed by Fang et al. on breast cancer models. They developed and characterized a new gene silencing approach: siRNA targeting CCL2 linked to TAT by non-covalent “soft” calcium bounds [73], which regulates the balance between TAT-siRNA complex stabilization and siRNA release after cellular uptake [74]. The chemokine CCL2 is overexpressed in invasive breast cancers and regulates their progression through multiple mechanisms [75]. They show that Ca-TAT/CCL2 siRNA can be efficiently delivered in vitro to breast cancer cells and in vivo to mice bearing triple negative breast cancer cell (MDA-MB-231) tumors. The results indicate that CPP-cargo complexes inhibit growth tumor and metastasis, as well as cancer stem cell renewal and recruitment of M2 macrophages. Among many other promising targets for CPP-siRNA therapeutic strategies, we can quote Nrf2, a transcription factor which can provide cancer cells with a growth advantage and cause resistance to chemotherapy in many cancers, including lung cancers [76,77]. Cyclin B1, the regulatory subunit of cyclin-dependent kinase 1 (Cdk1), is another interesting target, as it is overexpressed in several cancer cells and plays a role in cancer cell survival and proliferation [78]. In their study, Morris et al. used a peptide nucleic acid (PNA) analog of anti-cyclin B1 linked to a CPP called Pep-3. They showed that the intra-tumoral injection of the drug into prostatic tumor-bearing mice inhibits tumor growth [79]. Of note, as described initially by Gait and his team, PNA are a good alternative to oligonucleotides as they are unaffected by cellular nucleases and have strong RNA binding ability [80]. Moreover, they lack negative charge, which facilitates CPP conjugation.

### 2.4. Delivery of Proteins

Last but not least, an emerging strategy for the treatment of cancers relies on protein therapy. Twenty years ago, Dowdy and colleagues made the observation that CPPs such as TAT could delivery large biologically active proteins (120kDa β-galactosidase) into several organs after intraperitoneal injection into the mouse, without crossing the BBB [81]. This important breakthrough opened the gate for the use of CPP-conjugated therapeutic proteins and vaccine peptides. In the past 10 years, many research groups have tried to use CPPs for the development of vaccine delivery systems [82]. The aim is to deliver antigenic peptides into any antigen presenting cell, such as dendritic cells, ensuing processing and presentation followed by induction of an immune response. For instance, Pouniotis et al. describe the use of Penetratin linked to cytotoxic T lymphocyte epitopes derived from ovalbumin or mucin-1 tumor-associated antigens. In their paper, they show that these complexes are able to induce a stimulation of CD4+ and CD8+ T cells in vitro. In vivo, the secretion of cytokines due to T cell response inhibits B16.OVA melanoma cell growth. Moreover, pre-immunization with Penetratin-OVA protects mice from a subsequent tumor challenge [83,84]. This year, Brooks et al.’s work highlighted the therapeutic potential of a CPP linked to mucin-1 and T-cell epitopes. In a tumor vaccination model, this peptide induces multiple immune responses and delays mucin-1 tumors in mice [85]. In the same period, Shahbazi and Bolhassani performed a comparison of six cell penetrating peptides for the delivery of HPV16 E7 antigen in the context of HPV. E7 is an oncoprotein constitutively expressed by HPV-infected cells, and as such, a therapeutic vaccine target. Data obtained indicate that CPPs Pep-1, Cady-2, p28, and hPP10 can mediate E7 antigen entry into tumoral cells and that immunization of mice induces a long-term protection against tumor challenge, especially in the p28-E7 group [86].

Interestingly, p28 itself presents an anti-tumoral activity; p28 holds its penetrating properties from a domain derived from the protein azurin (amino acids 50 to 67), called p18 [22]. The full-length peptide prevents p53 ubiquitination in cancer cells, and thus induces a post-translational increase of p53 [87]. This peptide is able to inhibit angiogenesis and cancer cell growth [42,87,88]. In addition, p28 has been characterized as a tumor-homing peptide, meaning that it preferentially enters tumor cells [22]. In fact, Noei and his team took advantage of this property to design a novel therapeutic strategy, based on the fusion of p28 to apoptin [89]. Apoptin accumulates in the nucleus of tumoral cells, where it induces apoptosis in a p53-dependent manner [90]. Noei et al. show that p28-mediated targeted delivery of apoptin allows a selective cytotoxicity towards breast cancer cells, while sparing normal cells.

In the development of therapeutic peptides, the modulation of protein-protein interactions (PPIs) is also receiving a great deal of attention. PPIs are involved in almost every cellular process and tidily regulate signaling pathways. Thus, targeting deregulated PPIs in cancer could be an efficient therapeutic strategy. Tian et al.’s work focuses on SET-PP2A interaction [91]. SET is an oncoprotein that inhibits PP2A activity, a well-known tumor suppressor [92]. They engineered two peptides composed of a CPP Mut3DPT, linked to either SET or PP2A interaction sites: Mut3DPT-SET and Mut3DPT-PP2A. Data suggest that these peptides can both disrupt SET-PP2A interaction and have an anti-tumoral effect in vitro and in vivo. The same research team generated a Ras/Raf interfering peptide linked to Mut3DPT [93]. Ras/Raf activation is involved in the development of lymphoid cancers. The results of the study indicate that their peptide can interfere with the Ras/Raf pathway and induce tumoral cell death in vitro in chronic lymphocytic leukemia cell lines and primary cells. In vivo, treatment with the peptide extends survival in mice xenograft models. Other groups have identified interesting protein-protein interactions that could be potential targets in cancer [94,95,96]. 10 years ago, we reported the great interest of targeting the anti-apoptosis clone-11 (AAC-11) protein activity [97]. AAC-11 is an anti-apoptotic protein overexpressed in many cancer cells and tissues [98]. Its interaction with apoptosis-related proteins is known to be involved in different mechanisms leading to the development and progression of cancer [89]. A particularly interesting protein is the nuclear factor Acinus, which plays a role in the DNA fragmentation involved in the apoptotic process, and in which interaction with AAC-11 abrogates this function [98]. Results obtained previously showed that AAC-11 also has a critical role in the sensitivity to apoptosis induced by antitumor agents [99]. In particular, this protein negatively controls E2F1-dependent apoptosis [98]. It was also demonstrated that AAC-11 increased the formation of metastases via the Erk pathway [100] and that it was involved in the immune system escape through the FGF-2 cellular survival pathway [101]. Interestingly, inactivating mutations within the LZ domain of AAC-11, which functions as a protein-protein interaction module, repeals its pro-metastatic and anti-apoptotic properties, indicating the importance of this LZ domain for the carcinogenic properties of AAC-11 [100,101]. Thus, blocking AAC-11 sites of interaction (an alpha-helical leucine-zipper domain) could potentially inhibit the downstream pathways involved in tumoral cell activity. Therefore, we designed RT53, a peptide composed of the CPP Penetratin linked to the leucine zipper domain of AAC-11 as a competitive inhibitor of AAC-11 [89]. Our results show that RT53 is a tumor-homing peptide, able to selectively induce cell death in cancer cells while sparing normal cells [92,93]. In vivo, RT53 inhibits the growth of both BRAF wild-type and V600E mutant melanoma xenograft tumors [93]. More recently, we demonstrated that RT53 can induce immunogenic cell death via the emission of danger signals and in a fibrosarcoma tumor vaccination model [102]. Hence, this peptide could represent a promising therapeutic approach for melanoma and other cancers patients, as it encompasses both anti-tumor and immunotherapeutic effects, while presenting a tumor-homing property.

## 3. CPP-Based Anti-Cancer Therapy Optimization

As stated before, clinical applications of CPPs depend on the improvement of important parameters: the delay of degradation of CPPs by enzymes circulating in the plasma, the endosomal escape efficiency, and the improvement of cell/tissue-specificity.

### 3.1. Chemical Modifications to Enhance Therapeutic Delivery and Stability

Although the sequences of CPPs are rather variable, some structural features are commonly retrieved. For example, CPPs are generally composed of positively charged amino acids, with an advantage of arginine residues over lysines for internalization due to the presence of a guanidium group [65,103,104,105,106]. They also often display α-helical regions which have been shown to enter cells more effectively [107]. An optimal size and hydrophobicity are also important factors. Because of these known elements, it is now easier to predict the ability of a short peptide to penetrate cell membranes, using computational approaches, such as Hällbrink/Hansen et al., algorithm [108,109], or the AAN modeling method [110].

Several research groups focus on the synthesis of novel CPP sequences or the optimization of existing CPP sequences [104,111]. Their work consists of finding the shortest sequence necessary for cell entrance with the best delivery efficiency (cellular uptake and endosomal escape) and the best stability. This can be done by replacing some residues. For instance, in order to decrease sensitivity to cellular degradation, lysines can be replaced with unnatural ornithines [112], and l-amino acids can be replaced by d-amino acids [47]. It is possible to design protease-resistant CPP by the use of a shielding strategy. Indeed, positively charged CPPs can interact with negatively charged polymers, such as polyethylene glycol (PEG). Addition of PEG protects CPPs against degradation, bringing metabolic stability in the bloodstream and a longer biological half-time [113]. It is important to find the right balance between a good stability, insuring the delivery of the CPP-conjugated drug to the targeted cellular component, and its necessary elimination that prevents unwanted toxicity.

Another approach is to modify the structure of the CPP. For example, it is possible to enhance cellular uptake by changing peptides into cyclic peptides [51,114,115], dendrimers [116], or transform their side chains [117,118,119]. Furthermore, the addition of trifluoromethylquinoline moieties [120] or replacement of certain residues with histidines are common strategies to make endosomolytic CPPs [121,122]. As another option, Oskolkov et al. engineered stearylated TP10 analogs, named NickFects, with enhanced endosomal escape efficiency [123]. It is important to ensure that none of the peptides’ modifications alter their solubility, ease of synthesis, or toxicity.

Of note, the conditions and biochemical/structural modifications should be optimized individually according to the transduction task, due to a strong relationship between cell types, cargoes, and CPPs. Indeed, non-covalent cargo/CPP complexes are stabilized by common interactions between molecules, including polar, ionic, or hydrophobic interactions. Thus, different cargoes show different rank orders for “best” carrier. Moreover, it has been shown that the CPP’s mechanism of entry into cells is strongly influenced by the properties of the cargo. For instance, CPP TAT cellular uptake varies depending on the size of the cargo: for short peptides (less than 50 amino acids), a rapid and direct penetration occurs, while for bigger proteins (more than 50 amino acids), CPP-cargo are internalized via endosomal vesicles with slow rates [124].

### 3.2. Tumor-Homing CPPs

The most straightforward way to obtain specificity towards cancer cells is to develop tumor-homing CPPs. Biopanning of phage displayed peptide libraries on cells or tissues is one of the most common methods to identify such peptides. In this technique, the target cell type is exposed to a large combinatorial library of phages, which are modified in a way that their envelope can carry peptides of different lengths and structures. It is then possible to discriminate which phages associated to which peptides can bind or be internalized by tumoral cells and their normal counterparts. The strength of this method is that cell-specific peptides can be isolated without the prerequisite of knowing a surface biomarker [125,126]. In recent years, it allowed for identification of some tumor-homing peptides. For example, it has been found that the tripeptide motif Arg-Gly-Asp (RGD) was able to recognize specific integrins present at the surface of cancer cells [127]. Shortly after, it has been shown that coupling RGD-motif integrin ligands to chemotherapeutic agent doxorubicin allows for improvement of its efficiency towards humans breast cancer cells in vivo with limited toxicity [128]. Using the same technique, Zhou et al. found a novel CPP called MT23 with mouse melanoma cell specificity. MT23 can only enter B16 melanoma cancer cells and MT23-apoptin can significantly inhibit tumor growth and induce the cell apoptosis in B16 tumor-bearing mice [129].

The preference of some peptides for cancer cells has been discovered more randomly. For instance, BR2, which was initially designed as a shorter derivative of the anti-tumoral peptide buforin IIb, showed unexpected tumor-homing ability through interaction with ganglioside, via lipid-mediated macropinocytosis [130]. For its part, KRP is a basic polypeptide, which tends to accumulate in the acidic microenvironment of tumors. Then, cationic KRP is apt to adsorb to the negative charges of heparin D sulfate in the membrane of tumor cells through electrostatic interaction [67], while p28 and RT53 have also been shown to specifically target tumor cells [22,89]. The factor(s) responsible for this favorable property still remain to be investigated. It could be due to intrinsic features of tumoral tissues and cells or to the expression of a specific surface biomarker.

In addition, some groups have tried to engineer more complex delivery systems, conjugating a third molecule that confers specificity to the CPP-cargo composite. For example, Xiang et al. designed a novel tumor targeting drug delivery system by attaching a CPP called dNP2 to HPMA, a copolymer known to specifically target tumor tissues via the passive-targeting process (EPR effect) [131]. Results show that HPMA-dNP2-doxorubicine conjugates display higher and more specific uptake in cancer cells with enhanced activity compared to the drug alone.

### 3.3. CPP-Antibody Conjugates

Another strategy to improve specificity is based on tumor targeting using monoclonal antibodies (mAb). For example, Shin and colleagues designed a complex system composed on one side of a conjugate of heparin and a murine anti-CEA (anti-carcinoembryonic antigen) monoclonal antibody, and on the other side of a TAT-gelonin fusion protein [132]. CEA stands for anti-carcinoembryonic antigen, a receptor overexpressed at the surface of colorectal cancer cells. Gelonin is an inhibitor of protein synthesis with a strong cytotoxic effect. The two conjugates are able to associate through an electrostatic interaction between the cationic TAT and anionic heparin. The antibody specifically binds to CEA antigens overexpressed in the cancer cells. Once targeted, slowly released TAT-gelonin from the anti-CEA-heparin conjugate counterpart internalizes into tumor cells via TAT-mediated transduction, which results in apoptosis of the tumor cells. In vivo, treatment with the CPP-antibody conjugate induces a significant inhibition of tumor growth. This system, called “ATTEMPTS” (antibody targeted triggered electrically modified prodrug type strategy) has been declined in several other CPP-conjugated drugs and enables the drug to efficiently address potential specificity issues [133].

### 3.4. Activatable CPPs

Activatable cell-penetrating peptides (ACPP) are systems in which the CPP’s cell-penetrating function is masked with an anionic peptide by a cleavable linker. Once in the tumor tissue, proteolysis of the linker activates the cell-penetrating function of CPP [26]. Ingeniously, Tsien and his team took advantage of ACPP cleavable by matrix metalloproteinase-2 (MMP-2), known to be upregulated in most solid tumors, and to promote tumorigenesis. These ACPPs can target many xenograft tumor models from different cancer sites [134]. Similar strategies have been successfully used in several studies. For instance, in Cheng et al.’s experiments, the shielding group of 2,3-dimethylmaleic anhydride (DMA) is used to inhibit the cell-penetrating ability of the CPP (R8) at physiological pH 7.4. At tumor extracellular pH 6.8, the hydrolysis of DMA leads to charge reversal, activating the function of CPP to lead the cargo (doxorubicine) inside cells. Data showed that the ACPP exhibited significant tumor growth inhibition in vivo [135]. In another study, the ACPP is composed of a CPP (R8), an acid-labile linker (hydrazone), and a polyanionic domain. At pH 7.4, the positive charges of R8 are shielded by electrostatic interactions with the polyanionic domain. At pH 6.8, however, the inhibitory peptide detaches due to interaction breakage. Then, the unshielded CPP enters tumoral cells where it delivers its cargo, a polo-like kinase 1 siRNA that favors apoptosis [136]. There are numerous application examples of this novel ACPP system that take advantage of the tumoral microenvironment properties, in particular pH [26].

### 3.5. Passive Targeting with Intracellular Specificity

Finally, an interesting lead could be to rely on cellular mechanisms (deregulated pathways or conditions) unique to tumoral cells. In this strategy, there is no need for a cell-specific CPP. In practice, one example could be to take advantage of hypoxia, a phenomenon common to the majority of cancer cells. Hypoxia-inducible factors (HIFs) are the main effectors of cell response to hypoxia, by which they promote cancer cell survival and progression. Overexpression of ETD (ERK Targeted Domain) variants cause HIF-1 inactivation [137]. Thus, Karagiota and co-workers designed TAT-EDT peptides to target cancer cells under hypoxia in hepatocarcinoma models. Their data show a specific cytotoxic effect towards those tumoral cells, while producing no effects in normoxic cells [138]. In another strategy, Darwish and his team have designed a cyclic CPP conjugated with doxorubicine via disulfide bridge to construct a smart drug delivery system Dox-SS-CPP. The disulfide bridge is expected to increase the activity of the conjugate inside cancer cells because it can be cleaved with intracellular thiol, such as glutathione, which is more active in cancer cells compared to normal cells. Indeed, their results demonstrate an enhance cytotoxic effect of the conjugate towards cancer cells compared to the drug alone, with a limited toxicity [139].

## 4. Summary and Concluding Remarks

**Figure 2:**
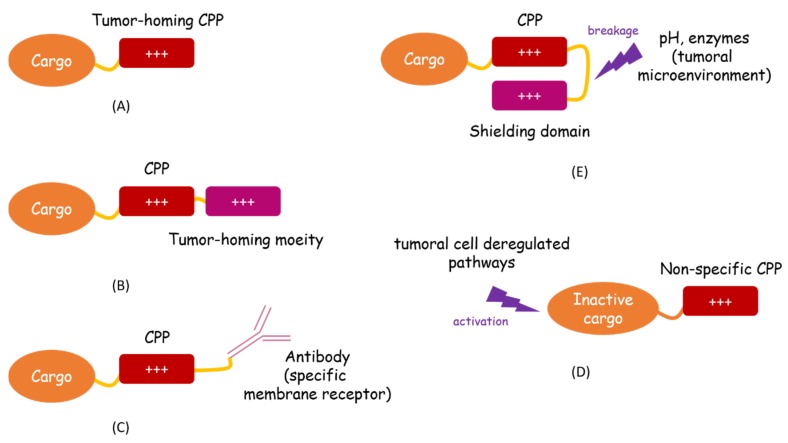
Strategies for tumor-specific CPP-conjugate delivery. To further enhance CCP-mediated intracellular uptake of conjugates, cargos can be linked to either tumor-homing CPPs (**A**), tumor-homing moiety (**B**) or membrane receptor specific antibody (**C**). Moreover, CPP-based drugs can be designed so they are only activated in the close neighborhood of tumor, where the microenvironment is different (**D**) or inside the transformed cell (**E**).

In the past 30 years, much effort has been dedicated to finding novel therapies against cancer. CPPs are receiving more attention thanks to their ability to deliver large cargoes of various nature inside cells. However, there is still no FDA (Food and Drug Administration) approved CPP-conjugated drug, although p28 is listed in two phase I trials (clinicaltrials.gov) for the treatment of solid p53 tumor [42]. The issues to address before translating CPPs into clinics are the following: the route of administration (oral administration is the best option for pharmaceutical industries); the stability in vivo and the non-specific intracellular uptake. Great progress has been made to improve those parameters. Indeed, many novel CPP-based delivery systems have been developed, introducing chemical and structural modifications or anti-proteases shielding for example. In the search for enhanced specificity, incredible advance has been made. Two main strategies have received particular attention: engineering peptides with a preference for tumoral cells or targeting cancer cell intracellular properties (summarized in Figure 2).

For the first strategy, the easiest way is to develop tumor-homing CPPs (Figure 2A). These peptides have a particular affinity for cancer cells or tissues because of physicochemical features of the tumor, or because of the expression of a specific biomarker. CPPs can also be coupled to a moiety that brings specificity towards tumoral cells (Figure 2B) or to an antibody that recognizes a specific marker expressed at the membrane of cancer cells (Figure 2C). Finally, it is possible to use ACPP—that is to say, shielding strategies, in which the CPP function is inactive in physiological conditions but activated in the close neighborhood of tumor, where the microenvironment is different (Figure 2D). For the second approach, CPP can enter any kind of cell but the cargo is only active inside tumoral cells, where molecular pathways are deregulated (Figure 2E). In terms of clinical applications, many factors need to be considered: cost, ease of synthesis, elimination, and immunogenicity, among others. Taking these elements into account, tumor-homing CPPs seems to be the most promising approach. Indeed, they are short and quickly eliminated, with a negligible toxicity. They are less expensive to produce than CPP-antibody conjugates. Moreover, the addition of complex molecules, such as antibodies, liposomes, nanoparticles, or biopolymers, increases the risk of immunogenicity. The same evaluation should be made concerning the cargo. Although coupling with a tumor-homing CPP appears to be a useful tool to reduce the chemotherapeutic agent’s toxicity, the risk of off-target effects, as well as development of resistance mechanisms, remain substantial. Application of gene therapy, while very promising in the case of inherited monogenic disorders, seems to be much more complicated in the context of cancer. The abnormalities are polygenic and there is high genetic heterogeneity, not only between tumors in different patients, but also between tumors at different sites within the same individual. On the contrary, targeting protein-protein interactions with CPP-protein conjugates allows for interference with many different pathways which are common to all tumors, with a limited risk of resistance-mechanism development. Stimulating the immune system with vaccine peptides also holds great promise. This technology allows the immune system cells to recognize tumor antigens more effectively and specifically attack and destroy cancer cells. Ultimately, peptides such as RT53 that combine (1) a tumor-homing property to (2) a specific effect on protein-protein interactions involved in cancer to (3) a specific immune response seem to be the most promising therapeutic strategy.

## Figures and Tables

**Table 1:** Classification of cell penetrating peptides.

Peptide	Sequence	Type	Lenght	Origin	References
Antennapedia Penetratin (43–58)	RQIKIWFQNRRMKWKK	Cationic and amphipatic	16	Protein-derived	Derossi et al., 1996 [7]
HIV-1 TAT protein (48–60)	GRKKRRQRRRPPQ	Cationic	13	Protein-derived	Green and Loewenstein, 1988; Frankel and Pabo, 1988 [8,9]
pVEC Cadherin (615–632)	LLIILRRRIRKQAHAHSK	Amphipatic	18	Protein-derived	Elmquist et al., 2001 [10]
Transportan Galanine/Mastoparan	GWTLNSAGYLLGKINLKALAALAKKIL	Amphipatic	27	Chimeric	Pooga et al., 1998 [11]; Langel et al., 1996 [12]
MPG HIV-gp41/SV40 T-antigen	GALFLGFLGAAGSTMGAWSQPKKKRKV	Amphipatic	27	Chimeric	Morris et al., 1997 [13]
Pep-1 HIV-reverse transcriptase/SV40 T-antigen	KETWWETWWTEWSQPKKKRKV	Amphipatic	21	Chimeric	Morris et al., 2001 [14]
Polyarginines	R(n); 6 < n < 12	Cationic	6–12	Synthetic	Wender et al., 2000 [15]
MAP	KLALKLALKALKAALKLA	Amphipatic	18	Synthetic	Oehlke et al., 1998 [16]
R6W3	RRWWRRWRR	Cationic	9	Synthetic	Delaroche et al., 2007 [17]
NLS	CGYGPKKKRKVGG	Cationic	13	Protein-derived	Ragin et al., 2002 [18]
8-lysines	KKKKKKKK	Cationic	8	Synthetic	Mai et al., 2002 [19]
ARF (1–22)	MVRRFLVTLRIRRACGPPRVRV	Amphipatic	22	Protein-derived	Johansson et al., 2008 [20]
Azurin-p28	LSTAADMQGVVTDGMASGLDKDYLKPDD	Anionic	28	Protein-derived	Taylor BN et al., 2009 [21]

**Table 2:** Selection of CPP-based therapies under clinical development.

CPP	Cargo	Application	Status	Compound	Company	ClinicalTrial.gov ID
R7	Cyclosporine A	Psoriasis	Phase II terminated 2003	PsorBan	CellGate. Inc	N/A
TAT	δPKC inhibitor	Heart attack	Phase II completed 2011	KAI-9803	KAI Pharmaceutical	NCT00785954
PTD4	HSP20 phosphopeptide	Wound healing	Phase II completed 2012	AZX-100	Capstone Therapeutics	NCT00825916
P28	P28	Central Nervous System Tumors	Phase I completed 2013	P28	Pediatric Brain Tumor Consortium	NCT01975116
P28	P28	Solid tumors	Phase I completed 2014	P28	CDG Therapeutics. Inc	NCT00914914
N/A	N/A	Duchenne Muscular Dystrophy (DMD)	Phase II completed 2015	AVI-4658	Sarepta Therapeutics	NCT00844597
TAT	JNK-1	Intraocular inflammation and pain	Phase III completed 2016	XG-102	Xigen SA	NCT02235272
TAT	JNK-1	Acute inner ear hearing loss	Phase III completed 2017	AM-111	Auris Medical	NCT02561091
MTS	Botulinum toxin A	Cervical Dystonia	Phase II completed 2018	R-002	Revance Therapeutics	NCT02706795
